# Relationships among inner strength, health and function, well-being, and negative life events in old people: a longitudinal study

**DOI:** 10.1007/s10433-021-00642-6

**Published:** 2021-10-18

**Authors:** Kerstin Viglund, Birgitta Olofsson, Berit Lundman, Astrid Norberg, Hugo Lövheim

**Affiliations:** 1 Department of Nursing, Umeå University, Umeå, Sweden; 2 Department of Community Medicine and Rehabilitation, Umeå University, Umeå, Sweden

**Keywords:** Cross-lagged panel model, Disease, Health, Inner strength, Longitudinal, Well-being

## Abstract

Inner strength is a conceptualization of a human resource that is generally considered beneficial for health and well-being. Previously, it has been examined in qualitative and cross-sectional studies, but longitudinal data are lacking. The aim of this study was to examine how inner strength, health and function, well-being, and negative life events, namely crises and diseases, affect each other over time in old people. A longitudinal two-wave design was used with data from 2010 and 2016. A total of 4023 participants, living in Finland and Sweden, and born in 1930, 1935, 1940, or 1945 were included. Data were collected using the Inner Strength Scale, the Life Orientation Scale, a short version of the Geriatric Depression Scale, one item from the SF36, and five items from the Katz ADL-index. Structural equation modeling was used to test for cross-lagged effects. Crises and diseases were found to be a positive predictor of inner strength, a negative predictor of well-being, and to have no significant effect on health and function over time. Inner strength and well-being had a reciprocal positive relationship, and health and function was a positive predictor inner strength. The study expands findings by providing perspectives of inner strength across time indicating that inner strength in old people increases when they have to face a disease or crisis. From a health perspective, the present findings reinforce the importance of healthcare professionals’ awareness and knowledge of the construct of inner strength.

## Introduction

Inner strength has been described as a human resource that promotes health and well-being, often in the context of life-threatening disease. Inner strength began to be examined in the healthcare literature in the 1990s. Reflecting on women’s psychological health, Rose (1990) described inner strength as “a paradoxical coalescence of vulnerability with safety, tenacity with flexibility, resolution with ambiguity, movement with stillness, and emotion with logic” (p. 61). Molony (1995) characterized inner strength as one’s ability to live through hard times and then move on. In accordance, Viglund et al. (2017) describe inner strength as one’s ability to draw strength from negative experiences. Meanwhile, Nygren et al. (2007) emphasize the benefits of living in a present that is connected to the distant past and future. Other descriptions of inner strength include having a positive view of life despite illness or loss (Mendes et al. 2010) and experiencing life-augmenting opportunities when old (Boman et al. 2015a). Inner strength has been suggested to enhance quality of life (Dingley and Roux 2014) and to enable nurturing through connection (Koob et al. 2002).

The inner strength literature consists mainly of qualitative studies with small samples and in large studies with cross-sectional designs. There are two research groups that have studied inner strength with the former approach (Smith et al. 2019). One of these groups, located in North America, has focused on health-related experiences of women with chronic diseases and developed a theory of inner strength based on findings from a meta-synthesis of findings from qualitative studies (Roux et al. 2002) and concept analysis (Dingley et al. 2000). The other group, located in Northern Europe, found significant correlations between salutogenic concept in relation to perceived physical and mental health among the oldest old and suggested that the concepts shared a common “area,” inner strength (Nygren et al. 2005). Lundman et al. (2010) developed a model of inner strength based on a meta-theoretical analysis of these salutogenic concepts. The European group has described inner strength as being composed of the four interrelated dimensions of firmness, connectedness, creativity, and flexibility. In a recent study, the term stretchability was used instead of flexibility (Lundman et al. 2019). Both research groups produced questionnaires based on their conceptualizations of inner strength (Lewis and Roux 2011; Lundman et al. 2011). The Inner Strength Scale (ISS) developed by Lundman et al. (2011) was used in the present study.

Studies with a cross-sectional design have shown that a higher degree of inner strength was associated with better health among older men and women (Viglund et al. 2013), and that inner strength is a partial mediator of the relationship between having a disease and self-rated health. That is, people with a higher degree of inner strength reported better self-rated health despite their disease (Viglund et al. 2014). In a group of older women, inner strength was found to be positively related to physical and mental health (Boman et al. 2017) and, conversely, negatively related to signs of depression (Boman et al. 2015b). Higher scores of inner strength showed an association with better subjective and objective health and more social contacts, in a group of very old people (Lundman et al. 2012). Inner strength, time since diagnosis, and comorbidity proved to be the strongest predictors of quality of life in a group of women with cancer (Dingley and Roux 2014), as well as overall health promoting behaviors among women with heart failure (Hosseini et al. 2016). Thus, estimates of inner strength have so far shown that higher degrees of inner strength have been significantly related to better health-related quality of life (Boman et al. 2017; Dingley and Roux 2014; Viglund et al. 2013), never or seldom feeling lonely, feelings of being needed, experience of having meaningful leisure activities (Boman et al. 2017), and being physical active (Hosseini et al. 2016).

Although inner strength has been examined since the 1990’s in relation to health, well-being, and disease, to the best of our knowledge, there are no longitudinal studies of inner strength. Therefore, it was a great opportunity to study inner strength over time with cohort data available from 2010 and 2016. Three hypotheses were tested based on previous research: (a) having a disease and/or going through a crises in life positively affects inner strength over time; (b) inner strength positively affects well-being over time; and (c) inner strength positively affects health over time. We used a cross-lagged panel model (CLPM) approach to provide more insight into and enhance the understanding of how old people’s inner strength may impact or be impacted by health, well-being, and management of diseases and crises. The aim of the present large longitudinal study was to examine in what ways inner strength, health and function, well-being, and negative life events including crises and diseases affect one another over time in old people. Data from time 1 in 2010 (T1) were compared to data from time 2 in 2016 (T2).

## Methods

### Design

Data were obtained from the Umeå 85 + /GERDA (Gerontological Regional Database) study, which examined older people’s health, well-being, and living situations in the northern Sweden and western Finland. In 2005, a survey was sent out to a cross-sectional sample of those born 1930 and 1940. Five years later, in 2010, the survey was sent out to people born in 1930, 1935, 1940, and 1945; these time data were coded to enable a follow up. Again, in 2016 the survey was sent out to the same sample as in 2010 to receive longitudinal data, as well as to people born in 1950. The present study was conducted with data from the 2010 and 2016 surveys.

### Participants and procedure

Potential participants were sent an invitation to participate in the GERDA study together with a survey. Their names, addresses, and civil registration numbers were collected from the National Tax Board in Sweden and the Population Register Centre in Finland. Briefly, every third person was selected randomly from the two largest cities in Västerbotten province (Sweden), which have approximately 120,000 and 72,000 inhabitants, respectively. Every second person was selected randomly from the largest city in the Österbotten region of Finland, which has approximately 67,000 inhabitants. In 2010, the GERDA survey was sent out to 10,969 people in Sweden and Finland, and 638 (62%) responded. In 2016, the GERDA survey was sent out to 14,805 people in Sweden and Finland, and 9386 (63%) responded. Finland is a bilingual country. Therefore, the survey was sent out in Swedish or Finnish, depending on the registered language of the respondent.

GERDA survey participants born in 1930, 1935, 1940 and 1945 were included in the present study, with the inclusion criteria of having completed the ISS in both 2010 and 2016. Of the 6119 participants who completed the ISS in 2010, 4696 completed the ISS in 2016 (dropout of 1423 persons, 23%, between the two time points). In the 2016 data, complete answers were missing in a number of cases, and mean scores were imputed for those missing less than 3 of the 20 ISS items. Therefore, there were a total of 4023 participants (86% of the 4696) with full ISS data from both 2010 and 2016.

### Measures

The ISS (Lundman et al. 2011) consists of 20 items, with 5 items for each dimension in Lundman’s Model of Inner Strength (Lundman et al. 2010): creativity (Crea), firmness (Firm), connectedness (Conn), and flexibility (Flex). The four dimensions were used as indicators of the latent construct of the ISS. The ISS is rated on a 6-point Likert-type scale, from “Totally disagree” (0) to “Totally agree” (6). Total ISS scores range from 20 to 120, with higher scores denoting higher degrees of inner strength. Cronbach’s alpha values obtained for the ISS in the present study (T1 alpha = 0.91; T2 alpha = 0.93) were similar to the 0.92 Cronbach’s alpha value reported by Viglund et al. (2014).

The Life Orientation Scale (LOS) (Pitkälä et al. 2004; Fagerström 2010) and a short version of the Geriatric Depression Scale (GDS) (Sheik and Yesavage 1986; D’Ath et al. 1994; Almeida and Almeida 1999) were used as indicators of the latent construct of *well-being* (WB). WB was based on how old people reported their experience of contentment with life, such as if they had a zest for life, if they had plans for the future, if they felt happy and satisfied, if they felt fear that something was going to happen, and if they felt depressed. The items were coded such that higher scores indicated greater WB. The LOS consists of six yes-or-no questions (1 point each) with a total score range of 0–6. We obtained Cronbach’s alpha values of 0.56 (T1) and 0.63 (T2) for the LOS. The GDS consists of four yes-or-no questions (1 point each) with a total score range of 0–4. We obtained Cronbach’s alpha values of 0.51 (T1) and 0.61 (T2) for the GDS. We obtained Cronbach’s alpha values of 0.69 (T1) and 0.67 (T2) for the LOS and the GDS together.

One item from the Short Form Health Survey (SF36) (Ware and Sherbourne 1992) and five yes-or-no items from the Katz ADL-index (ADL) (Katz et al. 1963, 1970) were used as indicators for the latent construct of *health and function* (HF). The SF36 item used asked, “In general, would you say your health is poor, fair, good, very good or excellent” with response scores ranging from 1 to 5. The included ADL items asked whether the participant was able to do each of five activities independently (showering, cleaning, grocery shopping, cooking, and management of public communication; 1 point each yes; total score range, 0–5). Thus, higher SF36/ADL scores indicated better health and function. Cronbach’s alphas of 0.63 (T1) and 0.70 (T2) were obtained for the ADL in this study.

For negative life events, a latent construct of *crises and diseases* (CD) was used. Nine negative life events were included in the crisis-index (Cri), and five diseases were included in the disease-index (Dis). Crises included in the Cri were own disease, relative’s disease, death in the family, and death of friends. The Cri items were dichotomous, answered with Yes (1) for having experienced a crisis during the last year or No (0) for not having experienced a crisis during the last year (Cri score range, 0–9). The diseases included in the Dis were stroke, myocardial infarction, diabetes, cancer, and pain. The Dis items were dichotomous, answered Yes (1) for having the disease or No (0) for not having it (Dis score range, 0–5).

### Missing data

In the 2016 data, among those not included in the analysis (*N* = 673), compared to those included (*N* = 4023), there were marginally more women, a higher percentage of people with the oldest birth year (1930), and a higher percentage of participants with a low level of education. Of the 4023 participants, the missing response rates for the LOS, GDS, ADL, and SF36 ranged from 0 to 12%, with the smallest missing response rate being for the SF36 (*N* = 25) and the largest missing response rate being for the GDS (*N* = 492). No impute of mean scores was made in these scales, and thus, only fully answered scales were included in the analysis (see numbers of participants in Table 2).

### Statistical analysis

**Fig. 1:**
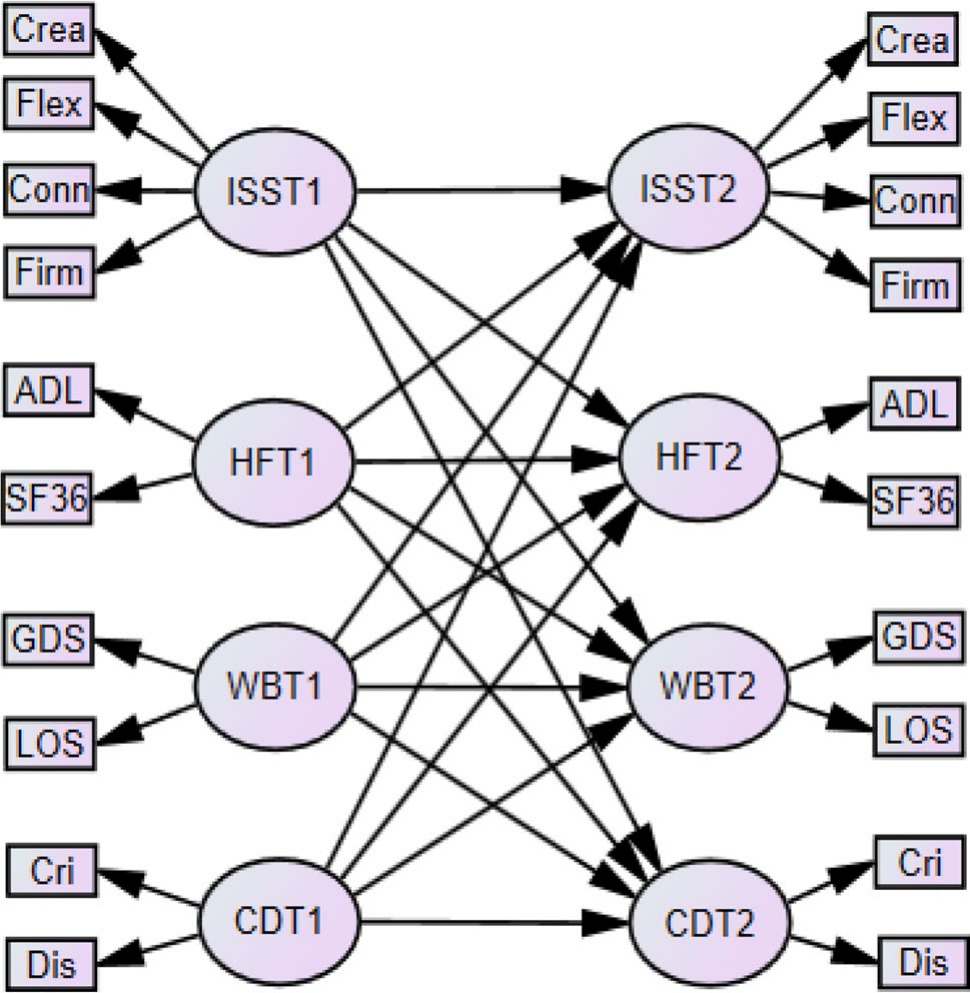
Simplified cross-lagged panel model with the four latent variables Inner Strength Scale (ISS), health and function (HF), well-being (WB), crises and diseases (CD), and the indicators for each latent variable, including creativity (Crea), flexibility (Flex), connectedness (Conn), firmness (Firm), activities daily life (ADL), Geriatric Depression Scale (GDS), Life Orientation Scale (LOS), crises (Cri), diseases (Dis)

Means are reported with standard deviations (SDs). Pearson correlation coefficients were used for assessing bivariate associations between variables. Paired sample t-tests were used, and Cohen’s d effect size calculations were used to test of mean differences across time points. Cutoff values were *d* = 0.2, *d* = 0.5, and *d* = 0.8 for small, medium, and large effects, respectively (Cohen 1988). Structural equation modeling with maximum likelihood estimation was used to detect autoregressive effects and cross-lagged effects. Autoregressive effects reflect stability from T1 to T2 of the four latent constructs ISS, HF, WB, and CD. A CLPM was used to examine how these four variables affect each other across T1 and T2 (Biesanz 2012). A simplified CLPM with the four latent constructs ISS, HF, WB, and CD was employed with the respective construct indicators shown in Fig. 1. Gender, age at T2, and education were used as control variables (not shown in Fig. 1).

Model fit was assessed by dividing the Chi-square value by its degrees of freedom (*x*^2^/df). The Chi-square test is sensitive to sample size (Fan et al. 1999) and this large sample study (*N* = 4023) had a significant Chi-square test result. We also used normal fit index (NFI), comparative fit index (CFI), and the root mean square error of approximation (RMSEA) to assess model fit. Cutoffs for a good model fit were NFI ≥ 0.95, CFI ≥ 0.95, and RMSEA ≤ 0.06 (Hu and Bentler 1999). SPSS 24.0 was used for descriptive and correlation analyses (IBM SPSS Statistics 2019), and AMOS 24.0 was used for structural equation modeling (Arbuckle 2014).

## Results

### Descriptive and bivariate statistics

The demographic characteristics are summarized in Table 1 together with comparisons of mean ISS scores at T1 (in 2010) and at T2 (in 2016) by gender, birth year, home country, and education level. Regarding the demographic makeup of the study sample, majorities of the participants were women and were from Sweden. Representation increased with each more recent birth year successively. Notably, mean ISS scores for all demographic classifications were very significantly reduced at T2, relative to T1, albeit with small effect size values. Effect sizes were progressively larger from younger to older age bands.

**Table 1:** Characteristics of the participants and comparisons of mean Inner Strength Scale (ISS) scores, with standard deviations (SDs), between assessment time 1 (T1) and 2 (T2) for each demographic classification

Characteristic	*N* (%)	Mean ISS score (SD)		*p* (effect size)
		T1 2010 *N* = 4023	T2 2016 *N* = 4023	
Gender				
Women	2159 (53.7)	100.3 (11.6)	98.1 (13.7)	< .001 (0.17)
Men	1864 (46.3)	98.9 (11.5)	97.1 (13.4)	< .001 (0.14)
Year of birth				
1930	380 (9.5)	98.0 (12.6)	94.0 (15.9)	< .001 (0.28)
1935	725 (18)	98.6 (11.4)	95.6 (14.8)	< .001 (0.23)
1940	1056 (26.2)	99.6 (11.9)	97.7 (12.7)	< .001 (0.15)
1945	1862 (46.3)	100.4 (11.2)	99.1 (12.7)	< .001 (0.11)
Country				
Sweden	2324 (57.8)	100.1 (11.8)	97.8 (13.8)	< .001 (0.18)
Finland	1699 (42.2)	99.0 (11.3)	97.4 (13.2)	< .001 (0.13)
Education				
Low	1679 (43)	98.6 (12.3)	96.2 (14.2)	< .001 (0.16)
Medium	1597 (40)	99.9 (11.0)	98.2 (13.1)	< .001 (0.14)
High	667 (17)	101.7 (10.8)	99.0 (12.5)	< .001 (0.23)

Bivariate correlation analysis showed that ISS scores correlated directly with SF36, ADL, LOS, and GDS scores at T1 and T2 (data reported in Table 2). Meanwhile, ISS scores tended to correlate inversely with Cri and Dis, except between T1 ISS scores and T2 Dis. SF36 scores had particularly strong negative associations with Cri and Dis. ISS, SF36, ADL, LOS, and GDS scores decreased from T1 to T2.

**Table 2:** Pearson coefficients of correlations among mean variable values, shown with standard deviations (SDs) and numbers of participants (N), at both time points

	1	2	3	4	5	6	7	8	9	10	11	12	13	14	15	16	17
1. Gender	–																
2. Age_T2_	− .05**	–															
3. Education	− .05**	− .12**	–														
4. ISS_T1_	− .06**	− .07**	.10**	–													
5. ISS_T2_	− .04*	− .13**	.10**	.49**	–												
6. SF36_T1_	.06**	− .15**	.13**	.20**	.20**	–											
7. SF36_T2_	.06**	− .22**	.12**	.18**	.24**	.61**	–										
8. ADL_T1_	− .23**	− .15**	.15**	.11**	.08**	.18**	.15**	–									
9. ADL_T2_	− .21**	− .27**	.12**	.13**	.19**	.24**	.31**	.48**	–								
10. LOS_T1_	.03	− .13**	.10**	.30**	.27**	.27**	.22**	.12**	.12**	–							
11. LOS_T2_	.05**	− .20**	.08**	.26**	.35**	.25**	.32**	.09**	.19**	.52**	–						
12. GDS_T1_	.05**	− .03	.00	.23**	.19**	.23**	.19**	.05**	.07*	.50**	.36**	–					
13. GDS_T2_	.10**	− .09**	.00	.19**	.27**	.22**	.28**	.07**	.15**	.36**	.56**	.48**	–				
14. Cri_T1_	− .10**	.06**	.00	− .07**	− .06**	− .20**	− .17**	− .03	− .03	− .16**	− .15**	− .21**	− .16**	–			
15. Cri_T2_	− .13**	.08**	.03	− .06**	− .08**	− .15**	− .23**	− .01	− .05**	− .08**	− .20**	− .10**	− .19**	.33**	–		
16. Dis_T1_	− .01	.10**	− .05**	− .05**	− .07**	− .39**	− .32**	− .10**	− .14**	− .10**	− .12**	− .11**	− .14**	.19**	.12**	–	
17. Dis_T2_	.04*	.10**	− .06**	− .03	− .06**	− .31**	− .36**	− .11**	− .18**	− .08**	− .13**	− .08**	− .14**	.16**	.17**	.58**	–
Mean SD				99.64 11.58	97.63 13.55	3.15 .99	2.93 .99	4.55 .90	4.14 1.26	5.46 .91	5.25 1.09	3.60 .74	3.54 .84	.69 .96	.75 .99	1.0 .77	.97 .88
N	4023	4023	4023	4023	4023	3995	3998	3826	3806	3736	3720	3531	3577	3968	3963	4004	4002

Gender: female = 1, male = 2. Age_T2_, in years: 70 = 1; 75 = 2; 80 = 3; 85 = 4. Education: low = 1; medium = 2; high = 3

*ISS* Inner Strength Scale, *ADL* activities daily life, *LOS* Life Orientation Scale, *GDS* Geriatric Depression Scale, *Cri* crises, *Dis* diseases

*p < .05; **p < .01

### Model analysis

The autoregressive model and the CLPM were both found to have a satisfactory goodness of fit (Table 3). The Chi-square test significance was attributable to the large sample size (*N* = 4023).

**Table 3:** Fit indices of the autoregressive model and the cross-lagged panel model (CLPM)

Model	*X*^2^(*df)*	*p*	NFI	CFI	RMSEA	90% CI of RMSEA
Autoregressive	2080.1 (192)	< .001	.936	.941	.049	.048–.051
CLPM	1953.9 (180)	< .001	.940	.945	.050	.048–.051

*df* degrees of freedom, *NFI* normal fit index, *CFI* comparative fit index, *RMSEA* root mean square error of approximation, *CI* confidence interval

**Fig. 2:**
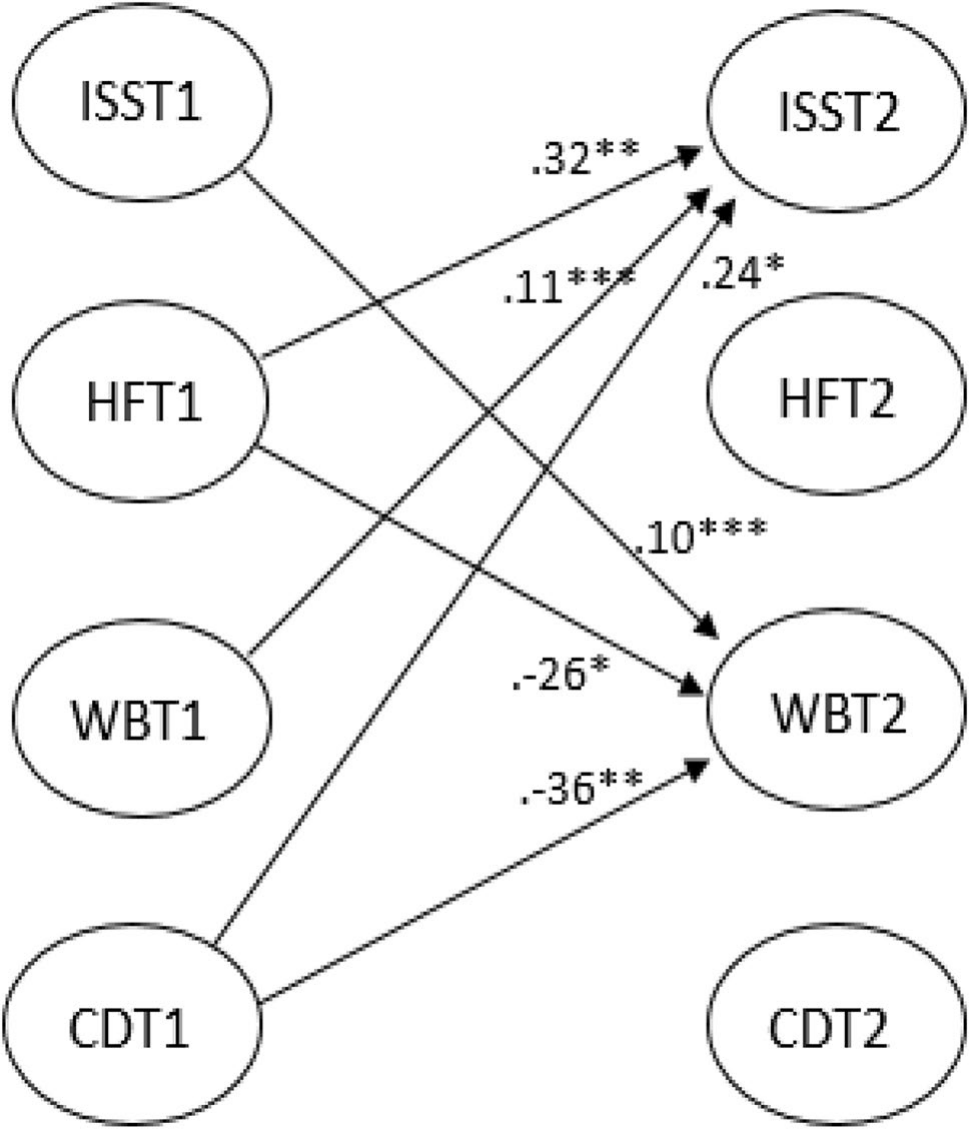
Cross-lagged path model (CLPM) with the latent variables Inner Strength Scale (ISS), health and function (HF), well-being (WB), and crises and diseases (CD) across time 1 (T1) and time 2 (T2). Only significant cross-lagged paths are indicated. *p < 0.05, **p < 0.01, ***p < 0.001

The standardized regression coefficients (*β*) of the autoregressive model and the CLPM are presented with p values in Table 4 (only significant cross-lagged paths in Fig. 2). All paths in the autoregressive model were highly significant, indicating stability over time for the latent variables. In the CLPM, high ISS scores at T1 predicted WB at T2 and, conversely, WB at T1 predicted ISS scores at T2. Better HF at T1 predicted greater ISS scores at T2, but ISS scores at T1 were not predictive of HF at T2. More CD at T1 was a positive predictor of ISS scores at T2, while more CD at T1 predicted worse WB at T2. Finally, better HF at T1 negatively predicted WB at T2.

**Table 4:** Standardized regression coefficients obtained for autoregressive model paths and cross-lagged panel model (CLPM) paths at time 1 (T1) and time 2 (T2)

Model	Autoregressive paths	*β*	Cross-lagged paths	*β*
Autoregressive	ISS_T1_ → ISS_T2_	.50***		
	HF_T1_ → HF_T2_	.78***		
	WB_T1_ → WB_T2_	.69***		
	CD_T1_ → CD_T2_	.79***		
CLPM	ISS_T1_ → ISS_T2_	.39***	ISS_T1_ → HF_T2_	.01
	HF_T1_ → HF_T2_	.92***	ISS_T1_ → WB_T2_	.10***
	WB_T1_ → WB_T2_	.61***	ISS_T1_ → CD_T2_	.05
	CD_T1_ → CD_T2_	.55***	HF_T1_ → ISS_T2_	.32**
			HF_T1_ → WB_T2_	− .26*
			HF_T1_ → CD_t2_	− .27
			WB_T1_ → ISS_T2_	.11***
			WB_T1_ → HF_T2_	− .05
			WB_T1_ → CD_T2_	.05
			CD_T1_ → ISS_T2_	.24*
			CD_T1_ → HF_T2_	.12
			CD_T1_ → WB_t2_	− .36**

*ISS* Inner Strength Scale, *HF* health and function, *WB* well-being, *CD* crises and diseases

*p < .05; **p < .01; ***p < .001

## Discussion

In the present longitudinal study designed to examine how inner strength, HF, WB, and CD affect each other over the 6-year interval from 2010 to 2016 in large samples of old people from Finland and Sweden, CD was a positive predictor of inner strength, as represented by ISS scores. Furthermore, CD was a negative predictor of WB, without a significant effect on HF over time. Inner strength and WB had a reciprocal positive relationship, whereas HF was found to be a positive predictor of inner strength.

Previously, researchers have often described experiences and perceptions of inner strength in relation to suffering from a severe disease (e.g., Mendes et al. 2010; Viglund et al. 2017; Tanaka 2018; Alexandersen et al. 2019), however, without being able to confirm causality. In the current longitudinal study CD predicted ISS, supporting the hypothesis that having a disease and/or going through a crises in life positively affects inner strength over time. Of interest may be that Waterworth et al. (2020) found that old people had difficulty identifying and discussing their strengths. The measure of inner strength used in this study is based on how old people describe their perceptions of their own connectedness to others (e.g., if they see themselves as part of a community, and if they can seek support from others when in a difficult situation). Our measure of inner strength also encompasses people’s perceptions of their ability to be creative and take on challenges, to carry out planned activities, and to be flexible and able to see things from different perspectives. Finally, the ISS used here queries respondents’ perceptions of themselves as people that can be trusted and that know their responsibilities (Lundman et al. 2010, 2011). It may very well be that it is difficult to find strength when it is most needed. From a nursing perspective, it is thus important that healthcare professionals be aware of and knowledgeable about inner strength so that they can provide support with relevant and appropriate interventions that can facilitate for patients who are affected by severe disease. Prioritizing the opportunities for patients to keep in touch with family and friends is one way of supporting connectedness. The Covid-19 pandemic has strongly demonstrated the need of being close and in regular contact with loved ones, in relation to health and quality in life among old people (see, e.g., Herrera et al. 2021; Strutt et al. 2021).

The bivariate correlation analysis showed that ISS scores were strongly related to the WB indicators of LOS and GDS scores. Additionally, in the CLPM, we found that ISS and WB scores were positive predictors of each other. That is, a high level of inner strength was associated with increased experience of well-being over time, and vice versa. At the same time, CD scores had opposing effects on ISS and WB scores over time. That is, increases in CD scores were associated with increased ISS scores and decreased WB scores. The result reflects the different meanings of inner strength and well-being, respectively, in relation to disease and crisis. In a large review study that examined aspects of subjective well-being (life satisfaction, happiness, sadness, and meaning in life) in relation to disease, the results indicated that disease states were predictive of impaired well-being (e.g., Steptoe et al. 2015). However, subjective well-being has also been found to be associated with improved survival among old people (Diener and Chan 2011; Steptoe et al. 2015).

A previous study indicated that inner strength may be a partial mediator of the relationship between disease and self-rated health among old people, such that those affected by disease may experience better self-rated health as a function of their inner strength (Viglund et al. 2014). In the present study, the result did not support the hypothesis that inner strength positively affects health over time. A possible explanation may be that the latent factor HF consisted of the indicators self-rated health (SF36 item) and ADL functions (Katz ADL-index). Self-rated health describes subjective experiences, while ADL functions describe objective facts that can be more difficult to influence, and ADL functions usually deteriorates with age. In addition, age had the greatest impact of the covariates while gender and education affected to some extent. Although the findings in these two studies are not directly comparable, there are some similarities between them. In the current study, CD was a positive predictor of inner strength, but was not significantly related to HF over time. Notably, both suggest that old people who have a disease or experience a crisis do not necessarily experience poorer health, though their inner strength tends to increase regardless. People have varying abilities to adjust to disease (Stanton et al. 2007; De Ridder el al. 2008), with inner strength being one factor that can contribute to one’s ability to adapt to disease worsening or a crisis. As mentioned, inner strength is thought to encompass creativity and flexibility in meeting such challenges. Healthcare professionals need to be aware of how to support patients’ creativity. It can be by paying attention and listening to their suggestions about their treatment and care. Many decisions are made without patient participation (Pel-Little et al. 2021). Supporting patients’ flexibility can be about, in dialogue with the person concerned, finding ways for him/her to adapt life to new or changed conditions.

Finally, in contrast to the positive relationship found between HF on inner strength, we obtained data indicating that HF may be negatively related to WB over time, such that healthier/more highly functional people report a lesser level of WB. It is possible that this reduction in subjective well-being is related, at least in part, to the fact that the study participants were 70–85 years old. At these ages, they were at an elevated risk of becoming ill or getting injured, while also be at risk for worsening of aging-related degenerative conditions, during the 6-year interval period (Christensen et al. 2009). However, the finding should be viewed cautiously, taking into account that there are mostly positive relationships between health and well-being described in the literature (e.g., Carmel et al. 2017; Kansky and Diener 2017), and we are not prepared to assign clinical importance to the results until more studies have confirmed them.

As far as we know, this is the first longitudinal study to examine inner strength in relation to WB, HF, and CD in old people. A strength of the study was its large sample size with participants from two Nordic countries, and relatively high response rates for both 2010 and 2016. It is likely that the age of the target population contributed to dropout between the two measurement occasions separated by 6 years. Missing data in 2016 can be attributed predominantly to the loss of the oldest participants, born in 1930, who also were most likely to have a lower education than participants born in later years.

When using a CLPM, as in this study, it is important to look for reciprocal effects or causality between variables; interacting indicators in the model need to be considered in the interpretation of the results (Biesanz 2012). The primary reason for using the four presently analyzed indicators (ISS score, HF, WB, and CD) was that they have been included in previous inner strength studies in various ways and to varying extents over the years. Therefore, we considered the inclusion of all four variables to be an advantage of this two-wave longitudinal study with a CLPM design. At the same time, we are aware that the areas of health, well-being, and disease are very extensive, and that we have touched a small part of the research field.

## Conclusions

The present study expands findings by providing perspectives of inner strength across time that has not previously been available. Inner strength, health and function, well-being, and crises and diseases in old people from Finland and Sweden were found to interact in various ways over a 6-year period. Crises and diseases were found to be a positive predictor of inner strength, a negative predictor of well-being, and to have no significant effect on health and function over time. Inner strength and well-being had a reciprocal positive relationship, and health and function was a positive predictor inner strength.

From a health perspective, the present findings reinforce the importance of healthcare professionals’ awareness and knowledge of the construct of inner strength. Inner strength is a complex construct, and the model of inner strength with its dimensions, connectedness, creativity, flexibility, and firmness, can be used as a guide to find interventions aimed to support old people dealing with a severe disease. So far, studies on inner strength have mainly focused on old people and on women. However, diseases and crises in life affects young people as well, and it would be meaningful to study inner strength among young people to get a lifespan perspective on inner strength.

## Data Availability

Data were obtained from the Umeå 85 + /GERDA (Gerontological Regional Database).
